# Obstructed Labor and Caesarean Delivery: The Cost and Benefit of Surgical Intervention

**DOI:** 10.1371/journal.pone.0034595

**Published:** 2012-04-25

**Authors:** Blake C. Alkire, Jeffrey R. Vincent, Christy Turlington Burns, Ian S. Metzler, Paul E. Farmer, John G. Meara

**Affiliations:** 1 Department of Otology and Laryngology, Harvard Medical School, Boston, Massachusetts, United States of America; 2 Program in Global Surgery and Social Change, Department of Global Health and Social Medicine, Harvard Medical School, Boston, Massachusetts, United States of America; 3 Nicholas School of the Environment and Sanford School of Public Policy, Duke University, Durham, North Carolina, United States of America; 4 Department of Population and Family Health, Mailman School of Public Health, Columbia University, New York, New York, United States of America; 5 Department of Global Health and Social Medicine, Harvard Medical School, Boston, Massachusetts, United States of America; 6 Department of Plastic and Oral Surgery, Children's Hospital Boston, Boston, Massachusetts, United States of America; University of Sydney, Australia

## Abstract

**Background:**

Although advances in the reduction of maternal mortality have been made, up to 273,000 women will die this year from obstetric etiologies. Obstructed labor (OL), most commonly treated with Caesarean delivery, has been identified as a major contributor to global maternal morbidity and mortality. We used economic and epidemiological modeling to estimate the cost per disability-adjusted life-year (DALY) averted and benefit-cost ratio of treating OL with Caesarean delivery for 49 countries identified as providing an insufficient number of Caesarean deliveries to meet demand.

**Methods and Findings:**

Using publicly available data and explicit economic assumptions, we estimated that the cost per DALY (3,0,0) averted for providing Caesarean delivery for OL ranged widely, from $251 per DALY averted in Madagascar to $3,462 in Oman. The median cost per DALY averted was $304. Benefit-cost ratios also varied, from 0.6 in Zimbabwe to 69.9 in Gabon. The median benefit-cost ratio calculated was 6.0. The main limitation of this study is an assumption that lack of surgical capacity is the main factor responsible for DALYs from OL.

**Conclusions:**

Using the World Health Organization's cost-effectiveness standards, investing in Caesarean delivery can be considered “highly cost-effective” for 48 of the 49 countries included in this study. Furthermore, in 46 of the 49 included countries, the benefit-cost ratio was greater than 1.0, implying that investment in Caesarean delivery is a viable economic proposition. While Caesarean delivery alone is not sufficient for combating OL, it is necessary, cost-effective by WHO standards, and ultimately economically favorable in the vast majority of countries included in this study.

## Introduction

In 2011, pregnancy-related complications resulted in an estimated 273,500 maternal deaths globally, or close to 775 deaths per day [1]. Regrettably, up to 90% of these deaths are preventable if diagnosed and treated in a timely manner [2]. Maternal mortality is a tragedy in any country, yet some face a much greater burden than others. Ninety-nine percent of maternal deaths occur in developing countries, and 65% occur in just 11 countries: Afghanistan, Bangladesh, the Democratic Republic of the Congo, Ethiopia, India, Indonesia, Kenya, Nigeria, Pakistan, Sudan, and Tanzania [3]. The majority of maternal deaths can be traced to five causes: postpartum hemorrhage, infection, obstructed labor leading to hemorrhage or infection, hypertensive disorders, and unsafe abortion [4]. Among these etiologies, one of the most common in developing countries is obstructed labor, defined by the World Health Organization (WHO) as labor in which “the presenting part of the fetus cannot progress into the birth canal, despite strong uterine contractions [5].” In the majority of cases, the risks posed by obstructed labor can only be averted by operative delivery of the fetus, which is most often by Caesarean delivery [5], [6], [7]. For the estimations and calculations in this study, obstructed labor (OL) is meant to indicate cases that are neglected or left untreated.

Premature death is not the only consequence of neglected obstructed labor: for every maternal death, there are many more cases of disabling sequelae, most commonly obstetric fistula [5]. An obstetric fistula is an abnormal communication between the rectum and vagina (rectovaginal fistula) or the bladder and vagina (vesicovaginal fistula). According to the United Nations Population Fund (UNFPA), as many as 3 million women are believed to suffer from obstetric fistula and another 30,000 to 130,000 cases develop each year in Africa alone [8]. Fistulas are devastating for the women who endure them: victims are often banned from their homes and shunned from their communities [8], [9]. Most affected women go without treatment for the duration of their lives despite the negative consequences on their health and social lives. More broadly, the effects of poor obstetric care in the developing world are not confined to the mother and child; the loss of productivity, destabilization of families and diversion of resources resulting from complications of OL amplify poverty within an individual household and the community at large [10].

Maternal death and obstetric fistula are preventable with timely diagnosis and treatment of obstructed labor, but this requires access to emergency obstetric systems, a service unavailable to most mothers in developing countries [11], [12]. Several studies have correlated the expansion of emergency obstetric services in developing countries with dramatically lowered rates of maternal mortality in the targeted regions [13], [14], [15]. With increased investment in health systems over the last two decades, the global maternal mortality ratio has decreased by 34% from its peak in 1990 [1], [3].

Despite the above-mentioned success in reducing maternal mortality, only 13 developing countries are on track to reach Millennium Development Goal (MDG) Five, which calls for a 75% reduction in maternal mortality ratios by 2015 [1]. Although international funding for maternal health has doubled in the last 5 years, it is still below the projected level necessary to reach MDG5 and is not aligned with countries that have the highest maternal mortality ratios [16]. Insufficient funding for maternal health remains a significant dilemma for international aid organizations and governments.

Quantifying the cost and benefit of averting the consequences of OL is essential for accurate analysis and informed health system funding prioritization [2], [10], [17]. The present study evaluates the impact of treating OL with Caesarean delivery in 49 countries, across multiple regions, identified by the WHO as providing an insufficient number of Caesarean deliveries to meet current demand [18]. Using previously published methodologies [19], [20] that estimate the number of disability-adjusted life-years (DALYs) averted secondary to a surgical intervention, convert said DALYs into estimates of economic impact for the affected populations, and compare the potential economic benefit with the cost of providing the surgical intervention, we determine a country-specific cost per DALY averted and benefit-cost ratio for providing Caesarean delivery in the context of OL. To our knowledge, this is the first study to quantify, in monetary terms, both the cost and benefit of treating OL with Caesarean delivery.

## Methods

### Study Cohort: Estimating the incidence of neglected obstructed labor and its sequelae

The relevant population in this study is the estimated number of women that incurred OL in 2008 in each of 49 countries noted by the World Health Organization as providing an insufficient number of Caesarean deliveries [18]. To estimate the number of cases of OL, we used a modeling approach based on publicly available retrospective data from the World Bank, U.N., and global burden of disease (GBD) study [21], [22], [23]. The GBD study [23] has included estimates of the incidence of OL and its sequelae since its inception. Our study relies on epidemiological estimates from the GBD study as the basis for our incidence and DALY calculations. Like the GBD study, we assume that left untreated, obstetric fistula is a permanent sequela once present in survivors of OL. Although the GBD study has traditionally included stress urinary incontinence (SUI) as a sequela of OL, recent evidence argues against a direct role of OL in the pathogenesis of SUI [24], and so we exclude SUI from our DALY estimates.

The epidemiological assumptions for this study are based on a GBD working paper from 2003, subsequent GBD reports, and systemic reviews [4], [5], [25], [26], [27], [28]. The GBD studies estimate the incidence of OL and its sequelae—obstetric fistula and maternal mortality—by WHO sub-region. Countries from the aforementioned WHO cost study were assigned to their appropriate WHO sub-region and subsequently assigned the incidence rates specific to the sub-region to which they belong. The WHO Caesarean delivery cost study included 54 counties; five countries included in the cost study—Azerbaijan, Kyrgyzstan, Tajikistan, Turkmenistan, and Uzbekistan—belonged to a sub-region in which the GBD study estimated 0.0 cases of OL. These countries are not included in our analysis.

Our estimates differ from the GBD studies as we only included women ages 15–49, as opposed to ages 15–59. We used the former as female fecundity is thought to approach 0% by age 45 [29]. Another key difference is that the GBD working paper [5] estimates a blanket incidence rate for all fertile women; since DALYs are sensitive to the age at which a patient incurs a disease process (due to age-weighting and changes in life-expectancy), efforts were made to apportion the incidence of OL and its sequelae (rectovaginal and vesicovaginal fistula, maternal death) according to seven female age-groups: 15–19, 20–24, 25–29, 30–34, 35–39, 40–44, and 45–49. This was accomplished by: i) calculating the total number of births in each of these age groups for every included country in 2008; ii) dividing the number of births in each age group by total births to calculate the relative proportion each age group contributes to total births; iii) calculating the total number of cases of OL and its sequelae for all women 15–49 using the incidence rates from the GBD working paper; and iv) multiplying the age-specific proportions from (ii) by the total number of cases of OL or its sequelae from (iii). An important limitation of this approach is that it does not account for the relationship between parity and obstructed labor: younger women are thought to have increased rates of OL because they are more likely to be nulliparous. Nevertheless, assigning different rates of OL to age groups by birth rate is still more granular than designating one rate for all women.

### Establishing the cost of a Cesarean Delivery

As part of a background report for the 2010 World Health Report, the World Health Organization (WHO) estimated the unit cost of a Caesarean delivery for 49 countries that were identified as providing an insufficient quantity to meet demand [18]. The inputs for estimating cost included “… initiation of labour at referral level, diagnosis of obstructed labour and referral, Caesarean delivery associated devices and medicines, operative facility time, medical human resources time, management of shock including hysterectomy and blood transfusion (assumed for 1% of CS performed), postoperative hospital stay for stabilization … programme administration, training, and the corresponding office space, electricity and other services, as well as a variety of standard consumables and equipment [18].” The estimated unit cost of a Caesarean delivery in each country is not explicitly stated in the report; instead, the total number of additional necessary Caesarean deliveries and total cost to provide said Caesarean deliveries are presented for each country. To obtain an average cost per Caesarean delivery for each country from this study, we divided the cost of supplying the necessary additional Caesarean deliveries by the number of additionally needed Caesarean deliveries to satisfy demand.

### Estimating disability-adjusted life-years (DALYs) and the economic benefits of treatment

A thorough overview of the DALY and the methods used to approximate the economic costs and benefits of surgical treatment are presented elsewhere (Appendix S1) [19], [20]. The following provides a brief explanation of the essential concepts and terminology that are necessary for understanding the methods and results of this study.

The DALY is a health metric that attempts to describe quantitatively the morbidity and mortality secondary to a disease process or risk factor in a population. One DALY is equivalent to the loss of one healthy year of life due to either early death or disability. Therefore, public health practitioners wish to avert DALYs in a population. To estimate the years lost while living with a disability, a predefined “disability weight” is multiplied by the number of years lived with the disability, where 0 = complete health and 1 = death. The benefit of a surgical intervention can be described in terms of the number of DALYs said intervention averts.

Importantly, not all Caesarean deliveries are meant to address OL, and not all cases of OL are treated with Caesarean delivery. To align with the GBD study, we estimated that 90% of OL cases require Caesarean delivery, while the remaining 10% can be addressed with instrumental vaginal delivery [5], [28]. These latter cases are excluded from our analysis.

In this study, we estimated the number of DALYs that could be averted in the 49 countries included in the WHO cost study if 90% of cases of OL were prevented in a timely fashion with a Caesarean delivery. Since Caesarean delivery is assigned a disability weight by the GBD study and carries a risk of mortality [30], we first calculated the gross number of DALYs that would be averted by preventing 90% of OL cases, and then subtracted the number of DALYs that would be incurred secondary to Caesarean deliveries to arrive at net DALYs averted.

DALYs can be calculated under different assumptions. The two most important ones pertain to discounting and age weights. Discounting the value of future DALYs to their present value is common practice and improves the economic comparability of DALYs that occur at different points of time. All of our DALY estimates are therefore discounted. We used a 3% discount rate, which has been used both in studies by the architects of the DALY concept [31] and in studies by experts on valuing mortality risk reductions [32]. The stated justification for age weighting in the DALY literature is that the social value of a year of healthy life is greater for young adults than for children or older adults. An age-weighting parameter, *β*, determines the age at which the DALY function peaks, with the peak occurring at 1/*β*. The most common value of *β* in the DALY literature is 0.04, which implies a peak at age 25. As described below, however, we used an alternative, country-specific age-weighting parameter, denoted by 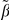, which is more consistent with empirical evidence on valuation of health risks.

To value a DALY in monetary terms, we adapted an economic concept known as the “value of a statistical life” (VSL). The VSL concept, grounded firmly in economic theory, uses empirical information on individuals' own valuation of the benefits of reducing the risk of premature death [33]. For example, a willingness to incur a cost of $100 in order to reduce the risk of death by 1/10,000 implies a VSL of at least $1 million ( = $100÷1/10,000). VSL estimates are obtained either from survey data or empirical wage data and are used in benefit-cost analyses by government agencies in the United States and other countries [34].

VSL studies have been performed in relatively few developing countries, but economists have devised a method for estimating the VSL in a country in which empirical studies have not been performed [35]. Using the ratio of gross national income per capita (GNI/capita) as a conversion factor, one can transfer the VSL from a higher-income country in which empirical studies have been performed to a lower-income country in which they have not. The key parameter in this transfer method is the “income elasticity of VSL” (IE-VSL), which determines how VSL changes in proportion to the relative income of the two considered countries. As IE-VSL increases, the transferred VSL estimate in the lower-income country decreases. Values of IE-VSL for transfers between developed and developing countries are typically in the range of 0.5–1.0, although some recent evidence suggests that even higher values are more appropriate for transfers to very low-income countries [36]. To err on the side of not exaggerating the benefits of addressing OL, this study uses an IE-VSL value of 1.5 for each included country.

Although VSL is defined in reference to reduced mortality risk, government agencies in the U.S. and abroad have used it to value decreased morbidity as measured by averted DALYs or their mirror image, quality-adjusted life-years (QALYs) [37], [38]. Health interventions such as Caesarean delivery for OL address both mortality (decreased maternal death) and morbidity (decreased rates of obstetric fistula), and so an analysis that valued only mortality reduction would underestimate the benefits of the intervention. Valuing averted DALYs using the VSL approach requires converting VSL to its annualized equivalent, the “Value of a Statistical Life-Year” (VSLY). Recent evidence on how individuals of different ages value reductions in mortality risks indicates that the VSLY changes with age, peaking at about 2/3 of life-expectancy [32]. To create consistency between DALYs and the VSLY estimates used to value them, we modified the DALY formula so that the age-weighting function peaks at 2/3 of a country's life expectancy, not a fixed 25 years as is the case with the commonly used *β* = 0.04. This modification is indicated by the notation 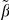, which signifies that we used country-specific age weights.

Like previous studies, we use the following notation to indicate the set of assumptions used in calculating DALYs: DALYs (r,K,*β*), where r = the discount rate, K = modulation of age-weighting formula (0 = age weights off, 1 = age weights on), and *β* = age-weighting parameter. For example, DALYs (3,0,0) indicates a 3% discount rate and no age weighting, while DALYs (3,1,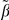) indicates a 3% discount rate and country-specific age weights.

### Estimating the Cost per DALY averted and Benefit-Cost Ratio

To estimate the total cost of providing the necessary number of Caesarean deliveries to prevent OL's sequelae, we multiplied the country-specific unit cost of a Caesarean delivery by the number of Caesarean deliveries required to treat 90% of the cases of OL in that country. Once the total country-specific cost was calculated, we divided said cost by the total number of DALYs (3,0,0) that Caesarean delivery for OL was estimated to avert. We chose DALYs (3,0,0) to allow our estimates to be compared to the interventions listed in *Disease Control Priorities in Developing Countries: 2^nd^ Edition* (DCPP) [39]. To calculate the benefit-cost ratio, we divided the country-specific economic benefit of treating OL by the total cost of providing the Caesarean deliveries required to do so, where the estimated benefit was based on DALYs (3,1,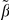).

## Results

### Estimated Incidence of OL and its Sequelae in 2008

A total of 3.1 million cases of OL are estimated to have occurred in the 49 countries included in this study in 2008. For each country, Table 1 presents the estimated number of Caesarean deliveries necessary to treat 90% of OL, along with the number of cases of obstetric fistula and maternal death that would be prevented by providing Caesarean delivery for OL. Maternal mortality is adjusted to account for mortality secondary to Caesarean delivery. For the 49 countries, an estimated 2.8 million Caesarean deliveries would have prevented 59,100 cases of obstetric fistula and 16,800 maternal deaths.

**Table 1:** Estimated number of Caesarean deliveries required to prevent 90% of OL in each country, along with the total number of preventable obstetric fistulas and maternal mortality for each country in 2008.

Country	Caesarean Deliveries a	Preventable Obstetric Fistulas	Preventable Maternal Mortality b
Algeria	54,600	1,160	690
Bangladesh	170,800	3,670	420
Benin	10,800	230	140
Burkina Faso	20,000	430	250
Cambodia	3,700	80	0
Cameroon	24,900	530	320
Central African Republic	5,300	110	70
Chad	13,200	280	170
Comoros	900	20	10
Côte d'Ivoire	22,800	480	310
Democratic Republic of the Congo	72,600	1,540	980
Eritrea	6,500	140	90
Ethiopia	97,800	2,070	1310
Gabon	2,000	40	30
Ghana	31,200	660	400
Guinea	12,000	260	150
Haiti	6,500	140	(10)
India	1,315,700	28,230	3220
Indonesia	83,400	1,730	320
Kenya	48,700	1,030	660
Lesotho	2,800	60	40
Liberia	4,700	100	60
Libyan Arab Jamahiriya	1,400	30	0
Madagascar	25,000	530	320
Malawi	16,200	340	220
Mali	18,000	380	230
Mauritania	4,500	100	60
Mongolia	500	10	0
Morocco	30,500	640	150
Mozambique	27,800	590	370
Nepal	31,900	690	80
Niger	17,200	370	220
Nigeria	190,200	4,050	2420
Oman	500	10	0
Pakistan	144,900	3,050	710
Philippines	10,300	250	0
Rwanda	13,000	280	170
Senegal	15,700	330	200
Sierra Leone	7,600	160	100
Sudan	33,900	710	160
Swaziland	1,500	30	20
Togo	7,900	170	100
Tunisia	2,400	50	0
Uganda	35,500	750	480
United Republic of Tanzania	50,300	1,070	680
Viet Nam	25,000	550	(20)
Yemen	18,000	380	90
Zambia	14,300	300	190
Zimbabwe	16,100	340	220
**Total**	**2,771,000**	**59,150**	**16,800**

a Necessary to prevent 90% of OL;

b Adjusted for mortality secondary to Caesarean delivery.

### Total DALYs Averted by Treating OL in 2008

Table 2 presents the total number of country-specific DALYs (3,0,0) and DALYs (3,1,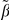) that would be averted if 90% of OL cases were treated by the provision of Caesarean deliveries in 2008. A total of 950,000 DALYs (3,0,0) or 1.1 million DALYs (3,1,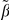) could be averted by providing the necessary number of Caesarean deliveries to treat OL. The similarity of these estimates indicates that the total number of DALYs averted is not very sensitive to the different assumptions about age-weighting.

**Table 2:** Estimated number of DALYs averted if the sequelae of OL were prevented by Caesarean delivery.

	DALYs averted	
Country	(3,0,0)	(3,1, 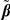 )
Algeria	29400	32700
Bangladesh	45700	51300
Benin	5400	6100
Burkina Faso	9900	11200
Cambodia	700	800
Cameroon	12000	13700
Central African Republic	2500	2900
Chad	6300	7200
Comoros	500	500
Côte d'Ivoire	11500	13100
Democratic Republic of the Congo	36900	41800
Eritrea	3600	4000
Ethiopia	50700	57000
Gabon	1000	1200
Ghana	16200	18100
Guinea	6000	6800
Haiti	1200	1400
India	346900	392700
Indonesia	24600	27700
Kenya	24400	27800
Lesotho	1200	1500
Liberia	2400	2700
Libyan Arab Jamahiriya	200	300
Madagascar	12800	14300
Malawi	7900	9100
Mali	8700	9900
Mauritania	2300	2600
Mongolia	100	100
Morocco	10500	11600
Mozambique	13100	15100
Nepal	8400	9500
Niger	8400	9600
Nigeria	90900	104000
Oman	100	100
Pakistan	47400	52900
Philippines	2700	3000
Rwanda	6900	7800
Senegal	8100	9100
Sierra Leone	3700	4300
Sudan	10700	12100
Swaziland	700	800
Togo	4100	4600
Tunisia	400	500
Uganda	17600	20100
United Republic of Tanzania	24400	28100
Viet Nam	5300	5900
Yemen	5800	6600
Zambia	6700	7800
Zimbabwe	6000	7500
**Total**	**952,900**	**1,079,500**

### The costs and benefits of providing Caesarean deliveries to prevent OL in 2008

Table 3 presents the total cost of providing the necessary number of Caesarean deliveries to prevent all cases of OL for the included countries in 2008, estimated by multiplying the total number of cases of OL (and thus the total number of Caesarean deliveries that need to be provided) by the unit cost of a Caesarean delivery. For each country, the total cost of providing Caesarean deliveries for OL was then divided by the potential DALYs averted if all cases of OL were treated to create a cost per DALY averted; as noted earlier, we used DALYs (3,0,0) in this calculation, to facilitate comparison to the estimates reported in DCPP. The cost per DALY averted varied by country, ranging from $251 to $3,462 per DALY. The median cost per DALY averted was $304.

**Table 3:** Unit cost of Caesarean delivery, total cost (USD) of treating OL with Caesarean delivery (000 s), cost (USD) per DALY(3,0,0) averted by treating OL, gross economic benefit of addressing OL (in 000 s), and benefit-cost ratio for treating OL in 2008.

Country	Unit Cost of Caesarean delivery	Total Cost (000 s) a	Cost/DALY averted b	Gross Economic Benefit (000 s) c	Benefit-Cost Ratio d
Algeria	$200	$10,930	$372	$738,500	67.6
Bangladesh	$98	$16,760	$367	$90,400	5.4
Benin	$142	$1,530	$281	$11,000	7.2
Burkina Faso	$140	$2,800	$284	$13,900	5.0
Cambodia	$148	$550	$743	$2,100	3.8
Cameroon	$144	$3,590	$298	$43,300	12.1
Central African Republic	$157	$830	$335	$1,800	2.2
Chad	$140	$1,840	$293	$8,000	4.4
Comoros	$141	$130	$273	$700	5.2
Côte d'Ivoire	$153	$3,490	$304	$26,300	7.5
Democratic Republic of the Congo	$131	$9,550	$259	$6,200	0.6
Eritrea	$139	$910	$254	$1,700	1.9
Ethiopia	$133	$12,980	$256	$46,900	3.6
Gabon	$361	$730	$709	$50,700	69.9
Ghana	$136	$4,240	$262	$32,800	7.7
Guinea	$130	$1,560	$258	$6,200	4.0
Haiti	$154	$1,010	$835	$1,600	1.6
India	$105	$137,580	$397	$2,060,400	15.0
Indonesia	$145	$12,060	$491	$190,500	15.8
Kenya	$139	$6,780	$278	$53,900	8.0
Lesotho	$202	$570	$469	$3,600	6.3
Liberia	$136	$640	$272	$500	0.7
Libyan Arab Jamahiriya	$498	$700	$2,886	$17,900	25.6
Madagascar	$128	$3,210	$251	$15,400	4.8
Malawi	$133	$2,160	$274	$5,800	2.7
Mali	$134	$2,420	$278	$12,100	5.0
Mauritania	$161	$720	$316	$7,200	9.9
Mongolia	$187	$90	$1,026	$700	7.1
Morocco	$169	$5,140	$488	$101,200	19.7
Mozambique	$137	$3,810	$291	$10,500	2.8
Nepal	$97	$3,090	$366	$11,200	3.6
Niger	$127	$2,180	$259	$5,600	2.6
Nigeria	$138	$26,330	$289	$290,100	11.0
Oman	$609	$320	$3,462	$12,300	38.8
Pakistan	$154	$22,300	$471	$221,800	9.9
Philippines	$152	$1,570	$589	$19,800	12.6
Rwanda	$137	$1,790	$259	$8,900	5.0
Senegal	$141	$2,220	$275	$21,900	9.9
Sierra Leone	$127	$970	$260	$2,900	2.9
Sudan	$156	$5,300	$495	$32,400	6.1
Swaziland	$226	$350	$508	$8,800	25.5
Togo	$132	$1,040	$255	$3,500	3.4
Tunisia	$350	$840	$2,017	$9,600	11.5
Uganda	$141	$4,990	$284	$24,300	4.9
United Republic of Tanzania	$139	$7,020	$288	$41,800	6.0
Viet Nam	$149	$3,730	$702	$26,400	7.1
Yemen	$156	$2,800	$480	$21,800	7.8
Zambia	$149	$2,120	$319	$10,500	4.9
Zimbabwe	$140	$2,260	$376	$1,300	0.6
**Median**	**$141**	**–**	**$304**	**–**	**6.0**

a : Total cost to treat 90% of cases of neglected obstructed labor with Caesarean delivery.

b : The cost per DALY averted using (3,0,0) assumptions, which is consistent with the DCPP approach.

c : Estimated by valuing DALYs (3,1,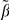) with value of a statistical life-year.

d : Benefit-cost ratio calculated by dividing gross economic benefit by total cost.

Table 3 also presents the country-specific gross economic benefit of preventing OL, using DALYs (3,1,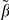). The total benefit across countries was estimated to be $4.3 billion. The last column of Table 3 shows benefit-cost ratios for providing Caesarean deliveries in each country, calculated by dividing the estimated economic benefit by the total cost of providing Caesarean deliveries. The benefit-cost ratio ranges from 0.6 for Zimbabwe to 69.9 for Gabon, with a median value of 6.0.

## Discussion

This study attempts to quantify the cost and benefit of treating neglected obstructed labor (OL) with Caesarean delivery. The global and regional burden of OL in terms of incidence, contribution to total deaths, and contribution to global DALYs has previously been described in the ongoing global burden of disease studies (GBD) [23], [26], [40]. As noted above, our study utilizes the epidemiological assumptions made by the GBD—with modifications to account for the fact that birth rates vary by age—to estimate the country-specific incidence of OL and its sequelae. For the 49 countries included in this study, 2.8 million cases of OL in 2008 were estimated to result in 59,100 cases of vesicovaginal or rectovaginal fistula and 16,800 maternal deaths. Given that these sequelae can be prevented with timely diagnosis, referral, and access to Caesarean delivery, these figures, although already known in global and regional terms, remain striking.

We also present an estimate of the total number of DALYs, calculated using the two sets of assumptions described above, that could be prevented with access to Caesarean delivery (Table 2). Without further context, these numbers have little meaning. However, when paired with the cost to prevent said DALYs with Caesarean delivery, a cost per DALY averted can be estimated, and comparisons can be made with other interventions with a known cost per DALY averted. Annex 2.B of the second edition of DCPP lists the cost per DALY (3,0,0) averted for more than 100 interventions, ranging from highly active anti-retroviral treatment for HIV positive individuals ($350-$1494/DALY averted in sub-Saharan Africa) to population-based immunization against rotavirus ($2,478–$2,945/DALY averted) [39]. Surgical interventions presented in DCPP include Trichiasis surgery ($39/DALY averted), cataract extraction ($180/DALY averted), and carotid endarterectomy for stroke prevention ($1,458/DALY averted). We estimate that Caesarean delivery for OL, depending on the country, costs $251 to $3,462 per DALY (3,0,0), with a median cost of $304 per DALY in the 49 countries included in this study. It thus compares favorably to the costs reported in DCPP; in fact, our median cost estimate is less than 46% of the cost per DALY estimates for interventions reported in DCPP.

The WHO, building upon work by the Commission on Macroeconomics and Health, has suggested thresholds for determining whether an intervention should be considered cost-effective: an intervention that costs more than three times the gross national income per capita (GNI/capita) per DALY is not considered cost-effective, an intervention that costs between one and three times the GNI/capita per DALY is considered cost-effective, and an intervention that costs less than the GNI/capita per DALY is considered highly cost-effective [17], [41]. By these standards, Caesarean delivery for OL is “highly cost-effective” in every country we examined except Zimbabwe, which still has a cost per DALY averted that qualifies as “cost-effective.”

Although economic analyses in the current global health literature are dominated by discussions of cost-effectiveness, there is growing interest in valuing improvements in health with measures that take into account the improvement in economic welfare that a longer, healthier life provides [39], [42]. Table 3 presents our economic valuation of potential DALYs averted by Caesarean delivery. Viewed in isolation, the estimates of the economic benefit of investing in Caesarean delivery for OL are large, but they are difficult to contextualize without being compared to the cost of the intervention. In a recent study, Jamison *et al.* also combined the potential cost of global health interventions with the potential economic benefit by valuing DALYs with a VSL approach, ultimately estimating benefit-cost ratios for seven interventions [42]. A key difference between their approach and ours, however, is that Jamison *et al.* assigned a blanket value of $1000 or $5000 to an averted DALY, based on the assumption that the VSLY is roughly 2–4 times per capita income. Our approach [Appendix S1] is more finely tuned to country characteristics, as it estimates a VSLY that varies with per capita income, life expectancy, age, and, if desired, the IE-VSL.

The benefit-cost ratios in Table 3 convey the fundamental finding of this study: the benefit-cost ratio is greater than one for every country we examined except Zimbabwe, Liberia, and Democratic Republic of the Congo; investment in Caesarean delivery will therefore not only reduce the tragic consequences of OL but will also yield an economic benefit that exceeds the cost. The median benefit-cost ratio for the 49 countries included in our study is 6∶1, which represents an excellent return on investment. Actual benefit-cost ratios might be even higher than we calculated, as we conservatively used the largest IE-VSL value ( = 1.5) reported in the literature, which reduces the estimated benefits. Even Zimbabwe, Liberia and Democratic Republic of the Congo have benefit-cost ratios greater than one if we instead use IE-VSL = 1, which is the most common value used in cross-country studies.

**Figure 1:**
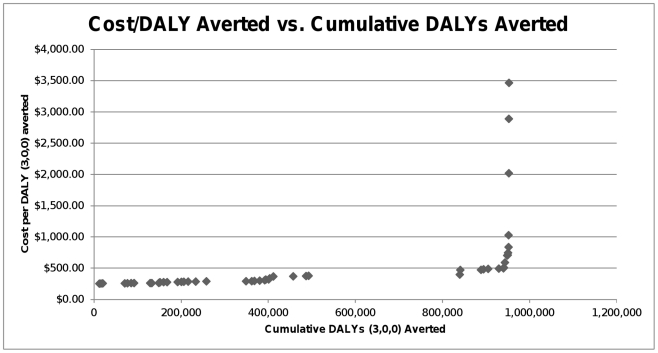
Cost per DALY (3,0,0) averted vs. cumulative DALYs (3,0,0) averted for the 49 countries included in the analysis. The vertical axis represents the average cost per DALY averted in a country, and the horizontal axis shows the cumulative number of DALYs averted as one moves from least-cost country to highest-cost country. To maximize the number of DALYs averted within their budgets, cost-conscious donors should first donate to countries at the left-most portion of curve and then move right until all funds are used.

Our study has a number of important implications for non-governmental organizations (NGOs), governmental organizations, and academicians. For potential donors and NGOs, we emphasize the relative cost-effectiveness of Caesarean delivery for OL in comparison to other interventions and WHO's per capita income thresholds. Our analysis is particularly useful if one is concerned with averting the most DALYs per dollar invested; the cost per DALY averted for each country can be ranked from lowest to highest and plotted against cumulative DALYs averted (Figure 1). Cost-conscious donors should begin by investing in the left-most country and moving progressively to the right until all funds are used. Our analysis further suggests that when prioritizing national funding priorities, governments should recognize that investment in healthcare can achieve net-positive economic benefits, as indicated by benefit-cost ratios greater than one. According to this metric, allocating sufficient annual funding for providing Caesarean deliveries within the broader context of maternal healthcare is an excellent economic proposition in almost all the countries we considered.

Our analysis has a number of important limitations. The most important is the implicit assumption that insufficient surgical capacity is main factor responsible for DALYs associated with 90% of OL. Our estimates of the benefits of Caesarean delivery to address OL are biased upward if, as the literature indicates, other factors matter, such as OL not being diagnosed in an accurate and timely manner or patients being unable to travel to clinics or hospitals for emergency obstetric care, as could be the case in remote regions with poor communications and transportation infrastructure [9], [43]. As noted earlier, the WHO cost estimates attempted to include costs related to “diagnosis of obstructed labour and referral,” but is possible that increased expenditure would not overcome barriers to improved diagnosis, referral, and access. The large benefit-cost ratios that we have estimated indicate, however, that surgical intervention would still be economically justified even if the actual number of Caesarean deliveries successfully performed were far fewer than the perfect rate we have assumed. At our median estimate of the benefit-cost ratio, surgical intervention would break even if only 1/6 of the potentially avertable DALYs were actually averted.

Strong evidence and a wealth of experience suggests that vertical programming of maternal health interventions, which Deborah Maine terms “one-complication programs” or “one-component programs,” are much less successful than interventions that aim to horizontally bolster health systems and thus address maternal mortality across the entire spectrum of obstetric care [44]. We agree, and have no intention of implying that Caesarean delivery alone is a magic bullet, or that an NGO should be formed to solely address OL. The current focus on packages of interventions, such as basic and emergency obstetric care, aim to ensure that women in need of an emergent Caesarean delivery are aware of facilities available to them, are properly diagnosed at the local level, are successfully transported to a referral hospital, and undergo safe Caesarean delivery in a capable facility [45], [46]. And yet there are still those who believe surgical interventions are too complex to implement, or that they require too great an investment to yield substantial gains in health [47]. In this broader context, our argument is that Caesarean delivery—as part of a larger strategy— can address maternal mortality in an economically favorable fashion.

The DALY's methodological imperfections and philosophical controversies are well-documented elsewhere [48], and a critique of our methodology for valuing DALYs is presented elsewhere as well [19], [20]. We recognize that there is uncertainty when estimating VSLs in low-income countries where formal studies have not been undertaken [36], but in choosing an IE-VSL of 1.5, we have minimized the risk of overestimation. Our estimates of cost per DALY averted (third column of Table 3) and benefit-cost ratios (last column of Table 3) are possibly too conservative (i.e., too high and too low, respectively), due to using the lower bound estimates of DALYs averted (3,0,0) for the former and the upper bound estimate of IE-VSL ( = 1.5) for the latter. Most importantly, we do not account for the reduction in perinatal mortality and morbidity that would undoubtedly occur with improved access to Caesarean delivery. Depending on the data source, the perinatal mortality rate secondary to neglected OL ranges from 38–92% [6], [7], [49].

Finally, our results are based on modeling of secondary data. Although our epidemiologic assumptions are based on the best available data, there is uncertainty regarding the true number of maternal deaths worldwide and the contribution that OL makes to that number.

While improvements in maternal healthcare have been well-documented [1], [3], there is still a chasm between the current reality and what is ultimately achievable. Although few would deny the horrific human consequences of neglected obstructed labor and the consequent need to combat OL for strictly humane purposes, the notion that surgical intervention is not cost-effective still exists. We demonstrate that investment in Caesarean delivery is cost-effective and can yield a net positive economic return within the context of a horizontally functioning health-system. The analyses utilized in this paper can be applied to other interventions and are crucial for better-informed investments in global health care delivery.

## Supporting Information

Appendix S1 (DOCX)
