# Supplementary material for: Obstructed Labor and Caesarean Delivery: The Cost and Benefit of Surgical Intervention
Source: PLoS One. 2012 Apr 25;7(4):e34595. doi: 10.1371/journal.pone.0034595 (PMC3338803; doi:10.1371/journal.pone.0034595)
Supplement: Appendix S1 — (DOCX) [file pone.0034595.s001.docx]

**Appendix S1**

*Calculating DALYs*

The basic formulas for calculating the number of DALYs due to incident disease in a population are:

DALYs = YLD + YLL,

YLD = I × D × DW

YLL = N × (LE-d)

where YLD = years lost due to disability, YLL = years of life lost, I = incident cases, D = duration of illness, DW = disability weight (0 = perfect health, 1 = death), N = total number of deaths due to disease, LE = life expectancy, and d = age of death. This study used the following disability weights from the GBD study [[1](#_ENREF_26)]: 0.430 for obstetric fistula, and 0.349 for Caesarean delivery.

Our study attempts to estimate the number of DALYs secondary to neglected obstructed labor. The above formula can be expanded to illustrate the specific variables examined in this paper:

DALYs (OL) = YLD (obstetric fistula) + YLL (maternal death due to OL)

To calculate the DALYs averted by providing Caesarean delivery for OL, we calculated the gross number of DALYs that would be prevented by treating OL and then subtracted the number of DALYs that would be incurred secondary to Caesarean delivery to arrive at net DALYs averted:

Net DALYs averted = [YLD (obstetric fistula) + YLL (maternal death due to OL)] –

[YLD (Caesarean delivery) + YLL (maternal death due to Caesarean delivery)]

Based on a recent WHO study of Caesarean delivery in 24 developing countries, we assumed a 0.15% maternal mortality rate secondary to Caesarean delivery [[2](#_ENREF_30)].

*Discounting and Age-weighting*

The inclusion of discounting and age-weighting results in a more complex DALY formula [3]:

(1)

where a *=* age of onset of disease*,* L = country-specific life-expectancy if calculating YLLs or the age at onset of a disease plus the duration of disease if calculating YLDs, K = age-weighting modulation constant (0 = no age weights, 1 = full age weights), DW = disability weight (1 for death), C = age-weighting correction constant, x = age integrated over the duration of disease (YLDs) or years of life lost (YLLs), r = discount rate (3% in this study), and *β* = age-weighting constant [[1](#_ENREF_26)].

For the special case of calculating DALYs to be valued using a VSLY approach, the formula is:

(2)

Compared to equation (1), the integral includes just one term because K =1 (age-weighting is turned on, because VSLY varies with age [[4](#_ENREF_32)]), which causes the second term to equal zero. The other key differences are the presence of $\tilde{\beta}$and $\tilde{\text{C}}$, where the tilde-mark indicates that country-specific age-weighting parameters and correction constants were used. Evidence indicates that VSLY peaks at about 2/3 of life expectancy [[4](#_ENREF_32)], so we modified the age-weighting factor in the DALY formula such that it peaks at 2/3 the life expectancy (LE) of each country included in this study. Since (1/*β*) = age at which the age-weighting factor peaks, to calculate country-specific *β*’s, we used the following expression to determine $\tilde{\beta}$ for each country:

$\tilde{\beta}$= 1 / [(2/3)·LE]

The value of C is also country-specific, as it varies with *β* according to Table 5.2 in the GBD [[1](#_ENREF_26)]. We fit a cubic polynomial to the values in that table and used it to predict $\tilde{\text{C}}$ for a given value of $\tilde{\beta}$.

*Converting DALYs averted to Economic Benefit*

To value DALYs using the VSLY approach, we first estimated the VSL in each country using the following formula [[5](#_ENREF_35)],

$$\text{VSL (Unknown)}=\text{ VSL (USA) }\cdot\left[\frac{\text{GNI p.c. (Unknown)}}{\text{GNI p.c. (USA)}}\right]^{\text{IE-VSL}}$$

where VSL(Unknown) = value of a statistical life in a country where VSL studies have not been performed, VSL(USA) = value of a statistical life in the United States ($7.4 million) [[6](#_ENREF_50)], GNI p.c.(Unknown) = GNI/capita in 2008 in a country where VSL studies have not been performed, GNI p.c. (USA) = GNI/capita in United States in 2008, and IE-VSL = the income elasticity of VSL. We used GNI/capita estimates based on the purchasing power parity (PPP) method [[5](#_ENREF_35)], and an IE-VSL of 1.5.

To calculate the potential economic benefit of an intervention that averts a given number of DALYs (3,1,$\tilde{\beta}$), we multiplied DALYs (3,1,$\tilde{\beta}$) by the value of a statistical life-year. VSLY_x_, the value of a statistical life-year at age x, is given by:

VSLY_x_ = V · $\tilde{\text{C}}$xe^-x^

where V= age-neutral (constant) value of a statistical life-year, and $\tilde{\text{C}}$xe^-x^ is the age-weighting factor found in the original DALY formula modified to peak at 2/3 of life expectancy. We discuss the calculation of V below. Note that using the DALY age-weighting factor creates internal consistency with the age-weighting of DALYs and the VSLY.

The formula for estimating the economic benefit of an intervention to the individual receiving it can therefore be written as:

Substituting the equation for VSLY_x_ into this results in the following:

If the constant V is moved out of the integral, the formula can be re-written as:

which by equation (2) reduces to:

$$\text{Economic Benefit }=\text{ V}\cdot\text{DALYs (3,1, }\tilde{\beta}\text{) }$$

The DALY formula already contains the age-weighting factor ($\tilde{\text{C}}$xe^-x^), and so we need only multiply DALYs (3,1,$\tilde{\beta}$) by V, not VSLY_x_, which would result in double age-weighting. Assuming one has already calculated DALYs (3,1,$\tilde{\beta}$), the only variable left to define is V, the age-neutral value of a statistical life-year. To solve for V, set DW = 1 and a = 0, which indicates that the disability is equivalent to death at birth. By definition, the economic benefit in this case is the VSL, and L = life expectancy (LE). Therefore, V is defined by the following expression:

Move the constants outside of the integral:

Solve for V and integrate:

As described above, multiplying V by DALYs (3,1,$\tilde{\beta}$) yields the economic value of averting these DALYs.

**References:**

1. Lopez AD, Mathers CD, Ezzati M, Jamison DT, Murray CJL, et al. (2006) Global burden of disease and risk factors. New York, NY; Washington, DC: Oxford University Press; World Bank. xxix, 475 p.

2. Souza JP, Gulmezoglu A, Lumbiganon P, Laopaiboon M, Carroli G, et al. (2010) Caesarean section without medical indications is associated with an increased risk of adverse short-term maternal outcomes: the 2004-2008 WHO Global Survey on Maternal and Perinatal Health. BMC Med 8: 71.

3. Murray CJ, Acharya AK (1997) Understanding DALYs (disability-adjusted life years). J Health Econ 16: 703-730.

4. Aldy JE, Viscusi WK (2008) Adjusting the Value of a Statistical Life for Age and Cohort Effects. Rev Econ Statist 90: 573-581.

5. Viscusi WK, Aldy JE (2003) The Value of a Statistical Life: A Critical Review of Market Estimates Throughout the World. J Risk Uncertainty 27: 5-76.

6. Environmental Protection Agency (2011) Frequently Asked Questions. Available: http://yosemite.epa.gov/ee/epa/eed.nsf/pages/MortalityRiskValuation.html. Accessed 2011 Oct 1.
